# Nitrogen deposition and management practices increase soil microbial biomass carbon but decrease diversity in Moso bamboo plantations

**DOI:** 10.1038/srep28235

**Published:** 2016-06-15

**Authors:** Quan Li, Xinzhang Song, Honghao Gu, Fei Gao

**Affiliations:** 1 The Nurturing Station for the State Key Laboratory of Subtropical Silviculture, Zhejiang A&F University, Lin’an, 311300, China

## Abstract

Because microbial communities play a key role in carbon (C) and nitrogen (N) cycling, changes in the soil microbial community may directly affect ecosystem functioning. However, the effects of N deposition and management practices on soil microbes are still poorly understood. We studied the effects of these two factors on soil microbial biomass carbon (MBC) and community composition in Moso bamboo plantations using high-throughput sequencing of the 16S rRNA gene. Plantations under conventional (CM) or intensive management (IM) were subjected to one of four N treatments for 30 months. IM and N addition, both separately and in combination, significantly increased soil MBC while decreasing bacterial diversity. However, increases in soil MBC were inhibited when N addition exceeded 60 kg N∙ha^−1^∙yr^−1^. IM increased the relative abundances of *Actinobacteria* and *Crenarchaeota* but decreased that of *Acidobacteria*. N addition increased the relative abundances of *Acidobacteria, Crenarchaeota*, and *Actinobacteria* but decreased that of *Proteobacteria*. Soil bacterial diversity was significantly related to soil pH, C/N ratio, and nitrogen and available phosphorus content. Management practices exerted a greater influence over regulation of the soil MBC and microbial diversity compared to that of N deposition in Moso bamboo plantations.

As the most abundant organisms on earth, microbes play a key role in natural ecosystems, including the biogeochemical cycles of carbon (C) and nitrogen (N) and the biodegradation or stabilization of environmental contaminants^1^,^2^,^3^. The soil microbial biomass has an important role in nutrient cycling and is therefore essential for plant growth^4^. Changes in the soil microbial community may directly affect soil ecosystem functioning, particularly C and N cycling^5^. In recent years, high-throughput sequencing technologies have been widely used to analyze the species composition and functional diversity of microbial populations under different fertilization regimes^6^,^7^,^8^. These technologies provide a more detailed description of microbial communities than traditional methods, such as cloning libraries, denaturing gradient gel electrophoresis (DGGE), and phospholipid fatty acid (PLFA) analysis and has proven to be a very powerful technique in microbial ecology research^9^. Because soil microbes are sensitive to environmental change^10^,^11^, a comprehensive analysis of the microbial community structure and microbial responses to environmental change using high-throughput sequencing technologies could improve our understanding of biogeochemical cycles in natural or managed ecosystems^12^.

Moso bamboo (*Phyllostachys pubescens* Mazel ex H. de Lehaie), distributed widely in subtropical China^13^, grows rapidly and is the most important source of non-wood forest products in China^14^,^15^. Under typical conventional management (CM), trunks and shoots of Moso bamboo are harvested regularly without any other management interventions^15^. In recent decades, intensive management (IM) practices, such as removing understory vegetation, plowing, and fertilization, have been widely adopted in order to maximize economic returns^13^,^15^. It has been reported that these IM practices alter the soil microbial biomass and community diversity^16^,^17^,^18^. For example, Liu *et al*.^19^ determined via polymerase chain reaction-denaturing gradient gel electrophoresis (PCR-DGGE) that fertilizer application significantly increased rhizosphere microbial diversity in a Moso bamboo forest. In contrast, Sun *et al*.^20^ observed that long-term conventional management of bamboo plantations did not significantly alter soil microbial biomass and diversity. However, few studies have used high-throughput sequencing technologies to assess the effects of management practices on soil microbial biomass and community diversity in Moso bamboo plantations.

Over the last three decades, the average bulk deposition of N has rapidly increased across China^21^, particularly in subtropical China^22^,^23^. Moso bamboo plantations are primarily distributed across the region with the greatest N deposition in China, according to both current estimates and future predictions^24^. Research has shown that N addition may reduce soil microbial biomass^25^,^26^ and community diversity^27^. Compton *et al*.^28^ and Frey *et al*.^29^ found that chronic N supplementation decreased soil microbial biomass at the Harvard Forest. However, Paul *et al*.^30^ found that the microbial biomass was greater under high N input (300 kg N∙ha^−1^∙yr^−1^) than under low N input (100 kg N∙ha^−1^∙yr^−1^). Johnson *et al*.^31^ also observed that N addition (80 kg N∙ha^−1^∙yr^−1^) increased the soil microbial biomass over seven years in a simulated N deposition study. Boxman *et al*.^32^ found that N deposition did not affect soil microbial biomass. The reasons for these differences in results remain unclear and require further study. The lack of understanding of the effects of N deposition on soil microbial quantity, community composition, and microbial diversity in Moso bamboo plantations with different management practices limits our ability to predict the complex response of plantation ecosystems to global environmental changes.

In the present study, we employed high-throughput sequencing to analyze the effects of nitrogen deposition and management practices on soil microbial community and diversity in Moso bamboo plantations. The objectives of this study were to test the following hypotheses: (1) IM practices increase soil microbial biomass carbon (MBC) and microbial diversity; (2) N deposition increases soil MBC and diversity; and (3) the combination of IM and N deposition exerts a greater effect on soil MBC and community composition than each practice independently.

## Results

### Soil microbial biomass carbon

**Figure 1:**
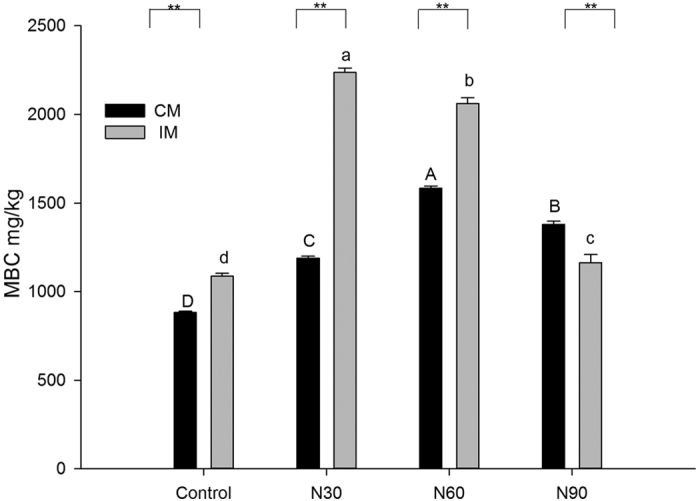
Soil microbial biomass carbon (MBC) contents under conventional management (CM) or intensive management (IM) and three N addition treatments.N30: 30 kg N∙ha^−1^∙yr^−1^; N60: 60 kg N∙ha^−1^∙yr^−1^; N90: 90 kg N∙ha^−1^∙yr^−1^. Lowercase letters indicate significant differences between N addition treatments under IM(*P* < 0.05). Capital letters indicate significant differences between N addition treatments under CM (*P* < 0.05).Asterisks indicate significant differences between CM and IM at identical N addition rates (*P < 0.05, **P < 0.01). Values represent means of three replicates, and error bars indicate standard errors.

Through assessment of soil samples from CM and IM plots in the Moso bamboo plantation, we found that soil MBC content was significantly higher under IM than under CM when no N was added (Fig. 1). Soil MBC content increased significantly with the addition of N under both CM and IM, but decreased significantly when the N addition rate exceeded 30 kg N∙ha^−1^∙yr^−1^ (N30 treatment) under IM and 60 kg N∙ha^−1^∙yr^−1^ (N60 treatment) under CM (Fig. 1).

In the IM plots, the maximum soil MBC content (2237.3 mg∙kg^−1^) was observed in the N30 treatment and was 105% greater than that observed in the control. In the CM plots, the maximum soil MBC content (1583.0 mg∙kg^−1^) was observed in the N60 treatment and was 79.6% greater than that in the control. While the soil MBC contents of the N addition treatments differed significantly from each other, all were significantly greater than that of the control treatment.

### Soil microbial community composition

**Figure 2:**
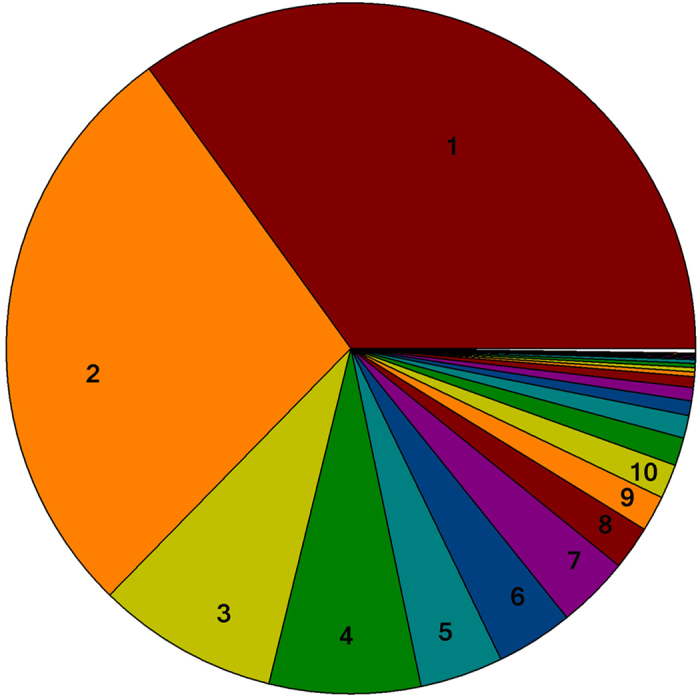
Taxonomic distribution of soil microbial communities in the Moso bamboo plantation under all treatments. Numbers indicate the ten most prevalent phyla: (1) *Proteobacteria*, (2) *Acidobacteria*, (3) *Verrucomicrobia*, (4) *Actinobacteria*, (5) *Chloroflexi*, (6) *Planctomycetes*, (7) *Gemmatimonadetes*, (8) *AD3*, (9) *Crenarchaeota*, and (10) *Others*, including sequences that could not be classified into any known group.

More than 35,000 valid reads were obtained for each replicate via a sequence optimization process and quality filtering. The median sequence length of each read was 253 bp. A total of 68,531 operational taxonomic units (OTUs) representing 41 phyla and 266 genera were detected using 97% identity as the cutoff. The dominant phyla were *Proteobacteria* (34.9%), *Acidobacteria* (27.7%), *Verrucomicrobia* (8.5%), and *Actinobacteria* (7%) (Fig. 2). In addition, 22 phyla were detected at relatively low abundances and accounted for less than 1% of the observed phyla. The dominant genera were *DA101* (4.3%), *Rhodoplanes* (1.8%), *Candidatus* Koribacter (1.7%), *Candidatus* Solibacter (1.4%), and *Rhodanobacter* (1.4%).

**Figure 3:**
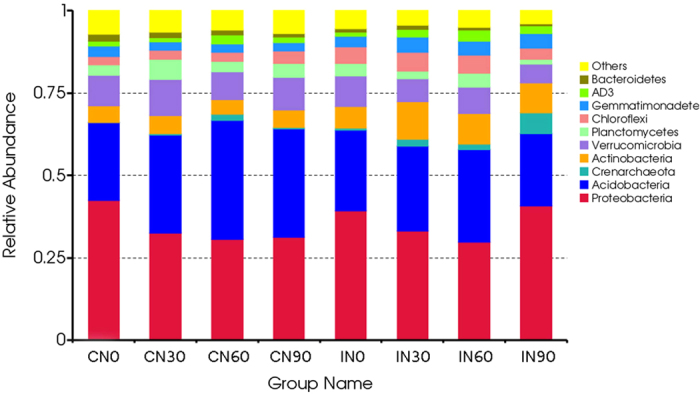
Comparison of the soil bacterial communities at the phylum level in the Moso bamboo plantation under all treatments. The relative abundances of the dominant bacterial groups in the soil differed depending on the nitrogen deposition and management practices. Relative abundances are based on the proportional frequencies of DNA sequences that could be classified. CN0: conventional management with no added N; CN30: conventional management with 30 kg N∙ha^−1^∙yr^−1^; CN60: conventional management with 60 kg N∙ha^−1^∙yr^−1^; CN90: conventional management with 90 kg N∙ha^−1^∙yr^−1^; IN0: intensive management with no added N; IN30: intensive management with 30 kg N∙ha^−1^∙yr^−1^; IN60: intensive management with 60 kg N∙ha^−1^∙yr^−1^; IN90: intensive management with 90 kg N∙ha^−1^∙yr^−1^.

Although fewer OTUs were detected in the CM plots (54,439) than in the IM plots (58,187), a greater number of phyla were observed under the CM treatments (39) than under the IM treatments (36). Under both CM and IM treatments, *Proteobacteria, Acidobacteria, Verrucomicrobia, Actinobacteria, Chloroflexi, Planctomycetes, Gemmatimonadetes, AD3, Crenarchaeota*, and *Bacteroidetes* contributed to a large proportion of phyla (Fig. 3). The relative abundances of *Actinobacteria* and *Proteobacteria* were higher under IM than under CM, whereas *Acidobacteria*, *Crenarchaeota*, and *Verrucomicrobia* exhibited the opposite trend.

No significant differences were observed in the number of OTUs among the N addition treatments. Under CM, N addition decreased the relative abundances of *Proteobacteria* and *Bacteroidetes* but increased the relative abundance of *Acidobacteria.* Under IM, N addition increased the relative abundances of *Crenarchaeota* and *Actinobacteria* but decreased the relative abundance of *Proteobacteria*, with the exception of the N90 (90 kg N∙ha^−1^∙yr^−1^) treatment.

### Soil microbial community and diversity

**Figure 4:**
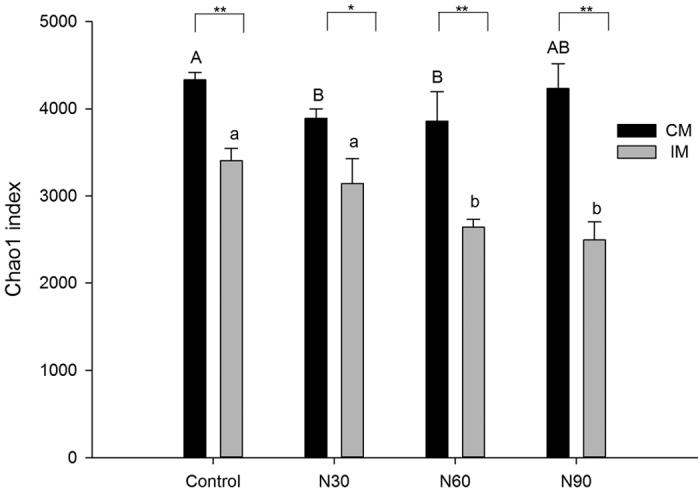
Chao 1 index of soil microbial diversity under conventional management (CM) or intensive management (IM) and three N addition treatments. N30: 30 kg N∙ha^−1^∙yr^−1^; N60: 60 kg N∙ha^−1^∙yr^−1^; N90: 90 kg N∙ha^−1^∙yr^−1^. Lowercase letters indicate significant differences between N addition treatments under IM(*P* < 0.05). Capital letters indicate significant differences between N addition treatments under CM(*P* < 0.05).Asterisks indicate significant differences between CM and IM at the same N addition rate (*P < 0.05, **P < 0.01). Values represent means of three replicates, and error bars indicate standard errors.

The Chao1 index, which reflects the species richness of a community, was significantly greater under CM than under IM treatment, regardless of N treatment. Index values were significantly lower in treatments with added N compared to that of the control under both CM and IM (Fig. 4).

**Figure 5:**
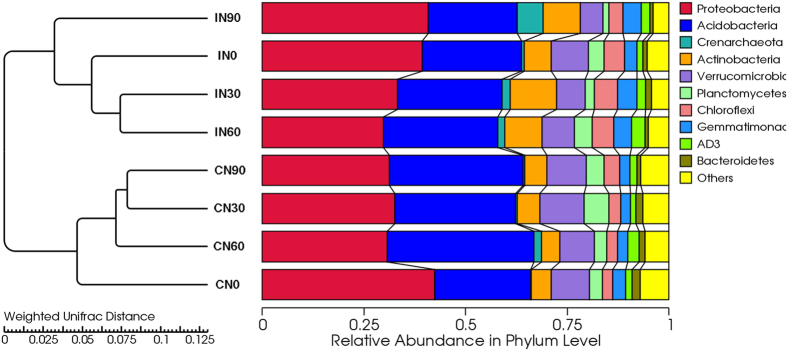
UniFrac UPGMA (unweighted pair group method with arithmetic mean) cluster analysis of microbial communities in soil samples from Moso bamboo plantation under different nitrogen deposition and management practice treatments. Figure was constructed based on Illumina sequencing data. CN0: conventional management with no added N; CN30: conventional management with 30 kg N∙ha^−1^∙yr^−1^; CN60: conventional management with 60 kg N∙ha^−1^∙yr^−1^; CN90: conventional management with 90 kg N∙ha^−1^∙yr^−1^; IN0: intensive management with no added N; IN30: intensive management with 30 kg N∙ha^−1^∙yr^−1^; IN60: intensive management with 60 kg N∙ha^−1^∙yr^−1^; IN90: intensive management with 90 kg N∙ha^−1^∙yr^−1^.

**Figure 6:**
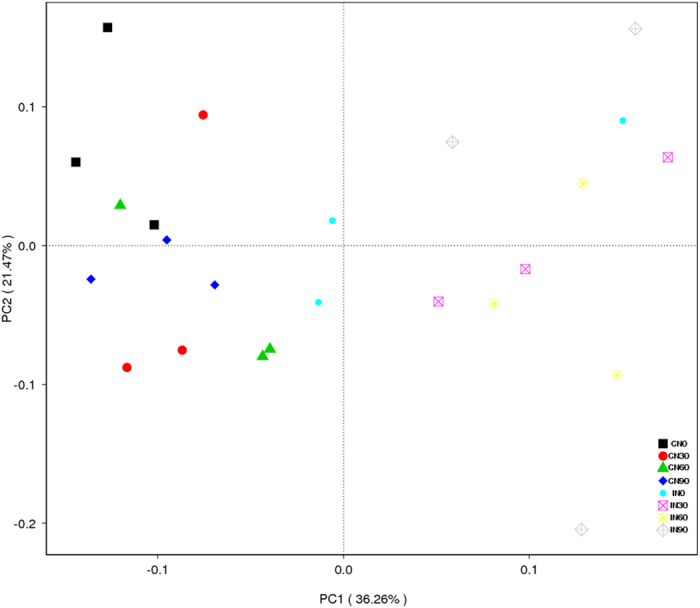
Principal coordinate analysis (PCoA) plot based on 16S rRNA gene sequencing of 24 samples. Scatter plot shows principal coordinate 1 (PC1) versus principal coordinate 2 (PC2). Percentages shown are percentages of variation explained by the components. CN0: conventional management with no added N; CN30: conventional management with 30 kg N∙ha^−1^∙yr^−1^; CN60: conventional management with 60 kg N∙ha^−1^∙yr^−1^; CN90: conventional management with 90 kg N∙ha^−1^∙yr^−1^; IN0: intensive management with no added N; IN30: intensive management with 30 kg N∙ha^−1^∙yr^−1^; IN60: intensive management with 60 kg N∙ha^−1^∙yr^−1^; IN90: intensive management with 90 kg N∙ha^−1^∙yr^−1^.

Cluster analysis of the soil microbial communities divided the bacterial communities into two groups (Fig. 5). All samples from the CM treatments clustered in group 1, and all samples from the IM treatments clustered in group II, regardless of N addition. This grouping was further confirmed by principal coordinate analysis (PCoA) (Fig. 6). Differences in microbial community structure were primarily due to a combination of nitrogen deposition and management practices (57.73%), with management practices alone accounting for 36.26% of the variation and nitrogen addition accounting for 21.47%.

### Relationship between microbial diversity and soil properties

**Figure 7:**
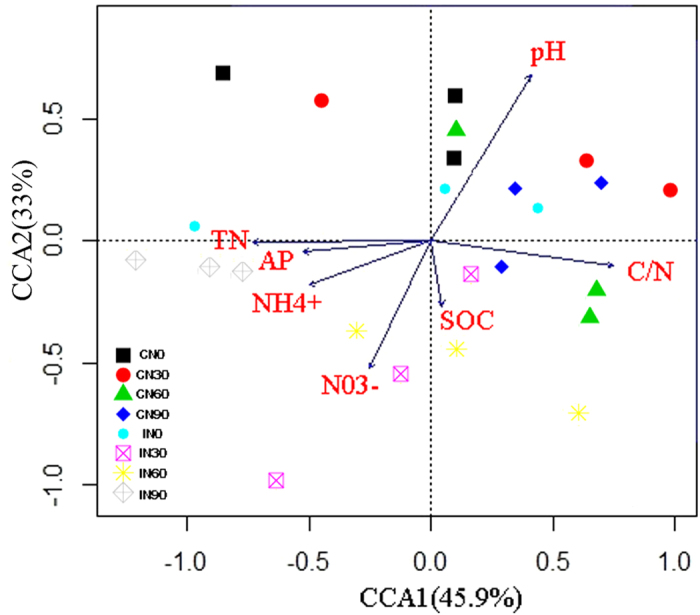
Canonical correspondence analysis (CCA) of the relative abundances of dominant microbial and soil environmental factors. Soil factors indicated in red text include pH, SOC (soil organic carbon), TN (total nitrogen), AP (available phosphorus), NH_4_^ + ^, NO_3_^−^, and C/N ratio. Arrow lengths indicate the strength of the relationship between the soil property and the overall microbial community. CN0: conventional management with no added N; CN30: conventional management with 30 kg N∙ha^−1^∙yr^−1^; CN60: conventional management with 60 kg N∙ha^−1^∙yr^−1^; CN90: conventional management with 90 kg N∙ha^−1^∙yr^−1^; IN0: intensive management with no added N; IN30: intensive management with 30 kg N∙ha^−1^∙yr^−1^; IN60: intensive management with 60 kg N∙ha^−1^∙yr^−1^; IN90: intensive management with 90 kg N∙ha^−1^∙yr^−1^.

The relationships between the *α*-diversity of soil microbes and soil properties were analyzed (Table 1). Chao1 and Shannon index values were significantly positively correlated with soil pH, and the Chao1 index was significantly negatively correlated with soil NO_3_^−^ and NH_4_^+^ concentrations. OTU number was also significantly positively correlated with pH but was negatively correlated with total N (TN), NO_3_^−^, and NH_4_^+^ concentrations. MBC was significantly negatively correlated with pH. Canonical correspondence analysis (CCA) demonstrated a correlation between primary soil properties and the microbial community; this showed that soil pH, C/N ratio, and available phosphorus (AP) and TN concentrations had the biggest impacts on the microbial community (Fig. 7).

## Discussion

### Effect of management practices on soil microbial biomass and community diversity

In this study, we found significantly higher levels of MBC in the IM treatments compared to the CM treatments without N addition (Figs 1 and 4), indicating that IM practices led to a significant increase in soil microbial biomass. This partially supports our first hypothesis, that IM practices increase soil MBC and diversity. IM practices such as fertilization may provide abundant nutrients for the growth of microorganisms and thus increase soil MBC^33^,^34^. Fertilization can also increase the productivity of Moso bamboo^35^, resulting in increased litter and nutrient return, which may contribute to microbial growth. Similar results were observed by Yu *et al*.^17^.

The second part of this hypothesis, that IM increases microbial diversity, was not borne out by the results. In fact, we found that IM significantly reduced soil microbial diversity. Similarly, He *et al*.^18^ observed that after 15 years of IM, the abundance of N_2_-fixing bacteria decreased in Moso bamboo forests. A comparable result was also observed in a *Castanea mollissima* forest^16^. Some management practices, such as plowing and weeding, may reduce the diversity of the aboveground plant community^32^, alter soil conditions, and destroy original habitat, potentially creating an unfavorable environment for some microbes and reducing microbial diversity. In addition, the osmotic potential in the soil solution of the IM plots may have become toxic due to the introduction of additional ions via fertilizer^36^, further reducing microbial abundance.

Previous studies found that fertilization changed the microbial community’s structure and composition^37^,^38^,^39^. Our results found that *Actinobacteria* were more prevalent with IM treatment, particularly with N addition. *Actinobacteria* can depolymerize the polyphenols in litter into small, soluble molecules and thus play an important role in the decomposition of lignin. The abundance of *Actinobacteria* therefore affects soil enzyme activity^40^. This result partially explains our previous finding that decomposition of leaf litter and lignin in Moso bamboo forests occurs more rapidly under IM than CM^24^. *Acidobacteria*, in contrast, were more prevalent in CM plots; these microbes are generally oligotrophic, consistent with their significantly lower abundances in the nutrient-rich rhizosphere and agricultural soils compared to that of bulk soil^5^,^41^. *Crenarchaeota* are a dominant group of microorganisms that govern NH_4_^+^ oxidation^42^. The decrease in the abundance of *Crenarchaeota* under IM that we observed may further affect the nitrifying process and N_2_O emissions in the soil of Moso bamboo forests.

### Effect of nitrogen deposition on soil microbial biomass and community diversity

Our results demonstrated that a low amount of added N increased the soil MBC, but when N addition exceeded a certain threshold (30 kg N∙ha^−1^∙yr^−1^ for IM and 60 kg N∙ha^−1^∙yr^−1^ for CM), a sharp decrease in soil MBC occurred (Fig. 1). This partially supports our second hypothesis, that N addition increases soil MBC and microbial diversity, but it is clear that the relationship is dependent on the amount of N added. We previously observed a limited amount of N in the soil of Moso bamboo plantations under IM, even though fertilization provided some N^43^. It is also known that N deposition can increase soil microbial biomass in an N-limited region^31^,^44^. Additional N input may directly increase available N and indirectly increase organic matter by enhancing plant productivity, thus promoting the growth of microorganisms^26^. However, excessive N input has negative effects on microbial activity. Many studies have reported that long-term nitrogen deposition reduced soil MBC^25^,^26^. Nitrogen saturation induced by excess N input can decrease soil pH, leading to leaching of magnesium and calcium and mobilization of aluminum^45^. When this happens, microbes may become magnesium- or calcium-limited or suffer aluminum toxicity^26^, resulting in reduced microbial biomass. Our results indicate that the N input threshold for Moso bamboo plantations may be 60 kg N∙ha^−1^∙yr^−1^ (Fig. 1).

As for the effect of N addition on microbial diversity, we found that added N significantly decreased soil microbial community diversity, particularly under IM (Fig. 4). Similarly, Frey *et al*.^29^ observed that the diversity of the ectomycorrhizal fungal community was reduced under N addition (50 kg N∙ha^−1^∙yr^−1^) compared to that of control areas in the Harvard Forest. Compton *et al*.^28^ also observed that N addition strongly influenced the DNA profiles of the microbial community. A meta-analysis revealed that the abundances of microbes decreased under N addition and that these reductions were more evident when higher total amounts of N were added and for longer durations^26^. In our study, the main reason for this reduced microbial diversity appears to be a reduction in soil pH induced by N fertilization, particularly with IM treatment. Previous studies have also found that fertilization treatment reduces soil pH^39^,^46^.

The N-induced reduction in microbial diversity may affect the structure of the microbial community^47^. CM is not conducive to nutrient accumulation, therefore, N addition resulted in a decrease in the relative abundances of eutrophic taxa (including members of the *Proteobacteria* and *Bacteroidetes*) and an increase in the abundances of oligotrophic taxa (mainly *Acidobacteria*) (Fig. 3). Under IM, the relative abundances of *Proteobacteria* and *Crenarchaeota* were higher in the N90 treatments than in the control, whereas *Acidobacteria* and *Verrucomicrobia* exhibited the opposite trend. Again, this may be because the relative abundances of eutrophic taxa (including members of the *Proteobacteria* and *Bacteroidetes*) increased, whereas those of oligotrophic taxa (mainly *Acidobacteria*) decreased in high-N plots^48^. Similar effects have been also observed in previous studies^28^,^49^. *Actinobacteria*, an important microbe in lignin decomposition^40^, exhibited a greater relative abundance in the N30 treatment than in the N90 treatment under both IM and CM, thus providing a reasonable explanation for our previous observation that a low level of N addition facilitates the decomposition of Moso bamboo leaf litter, while a high level suppresses decomposition^24^.

### Interactive effect of nitrogen deposition and management practices on soil microbial biomass and community diversity

Our third hypothesis was that the combination of management practices and N deposition would exert greater effects on soil microbial biomass and diversity than either practice independently. Two-way ANOVA demonstrated that both management practices and N deposition had significant positive effects on soil MBC (Fig. 1 and Table 2) but significant negative effects on soil microbial community diversity (Fig. 4 and Table 3). These patterns were true for the two variables, both alone and in combination, supporting our third hypothesis. PCoA and cluster analyses demonstrated that management practices had a greater impact on soil microbial diversity than nitrogen deposition (Figs 5 and 6). IM practices such as plowing, weeding, and fertilization alter the structural and physicochemical properties of soil more than the addition of nitrogen alone. Moreover, IM has been performed at the site for approximately 15 years, whereas the N addition experiment was only conducted for 2.5 years. The cumulative effects of IM practices on soil microbes were therefore greater than those of N addition owing to the short duration of the experiment.

### Relationship between microbial diversity and soil properties

Previous research indicated that soil pH may strongly influence the soil microbial community composition and bacterial diversity. Soil pH is thought to be a good predictor of bacterial community composition^28^,^37^,^39^,^50^,^51^,^52^,^53^,^54^. The results of this study demonstrated that bacterial diversity and community composition were mainly correlated with soil pH (Fig. 7 and Table 1). A strong negative relationship between bacterial diversity and soil pH was also observed by Lauber *et al*.^55^ and has been supported by other studies^8^,^50^. Our results also demonstrated that N, available P, and the C/N ratio are important parameters for shaping microbial community structure (Fig. 7). In accordance with this, previous studies observed that bacterial community structure was closely correlated with the NO_3_^−^ concentration^39^,^56^,^57^. Additionally, Zhao *et al*.^58^ observed that *Betaproteobacteria* and *Deltaproteobacteria* abundances were positively correlated with available soil P content. Chu *et al*.^59^ observed that the C/N ratio was significantly correlated with the relative abundances of various dominant phyla in Arctic tundra soils, consistent with the abundances and composition of soil bacterial communities in the Changbai Mountains^60^. These results indicate that there are significant correlations between the diversity and composition of microbial communities and soil properties. Therefore, management practices and N addition may affect microbial diversity through changes in soil parameters.

In the present study, measurement of the soil microbial biomass included both bacteria and fungi, whereas assessment of the V4 hypervariable region of the 16S rRNA gene only reflected changes in the bacterial community composition. A previous study revealed that N deposition also inhibits fungal community composition^26^. Therefore, future studies should explore the effects of N deposition and management practices on fungal diversity.

In conclusion, we found that while both intensive management and N addition increased soil microbial biomass and reduced microbial diversity on a Moso bamboo plantation, management practices exerted a greater influence over regulation of these parameters than N deposition. These results not only have practical implications for the management of Moso bamboo forests, but also serve to broaden our understanding of the effect of human interventions and N deposition on soil microbial communities.

## Methods

### Study site

The study site was located in Qingshan Town, Lin’an City (30°14′N, 119°42′E), Zhejiang Province, China. The area has a monsoonal subtropical climate with a mean annual precipitation of 1,420 mm and a mean annual temperature of 15.6 °C, ranging from 24 °C in July to 3 °C in January. The area receives an average of approximately 1,847 hours of sunshine per year and features an average of 230 frost-free days per year.

### Experimental design and measurement

Detailed information on the experimental design can be found in Song *et al*.^24^. Briefly, CM and IM Moso bamboo plantations were established at the study site. The CM plantation was originally established in the late 1970 s, and the IM plantation was established based on the CM area in 2001. CM practices included only the selective and regular harvest of bamboo trunks and shoots, which also took place in the IM area^24^. The IM area included additional management practices, such as plowing, weeding with herbicides, and fertilization. Specifically, in September of each year, 450 kg∙ha^−1^ of compound fertilizer (15:6:20 N:P_2_O_5_:K_2_O) was manually and evenly scattered on the soil surface, followed by deep plowing to 0.3 m. There was a greater abundance and biomass of plant species in the CM area than in the IM area^24^.

For this study, we established 12 CM plots and 12 IM plots in November 2012. Each plot had an area of 20 × 20 m and was surrounded by a 20-mbuffer zone. In accordance with the local background atmospheric N deposition rate of 30–37 kg N∙ha^−1^∙yr^−1^ 61,62,63, N addition treatments included a low-N treatment (30 kg N∙ha^−1^∙yr^−1^, N30), a medium-N treatment (60 kg N∙ha^−1^∙yr^−1^, N60), and a high-N treatment (90 kg N∙ha^−1^∙yr^−1^, N90). Three replicate plots per treatment were randomly placed in each management area. Beginning in January 2013, following a widely used method for simulating N deposition^64^,^65^, NH_4_NO_3_ was dissolved in 10 L water to the proper final N concentration and sprayed evenly on the forest floor with an electric sprayer at the beginning of every month. An equivalent amount of N-free water was sprayed on each control plot to control for the effect of the added water^24^.

### Soil sampling

In July 2015, ten soil cores at a depth of 0–20 cm were randomly collected from each plot and mixed together. Samples were sieved through 2-mm mesh to remove roots, plant residues, and stones. A portion of each soil sample was collected in a 50-mL centrifuge tube, placed in a cooler, and transferred to the laboratory. The tubes were archived at −80 °C until being used for molecular analysis. The remaining samples were used to measure the microbial biomass (from field-moist soil) and were then air-dried to determine the physicochemical properties of the soil.

### Analysis of soil microbial biomass and physiochemical properties

MBC generally accounts for 40–50% of the microbial biomass and 1–5% of soil organic carbon, and is thus regarded as an important indicator of soil microbial biomass^66^. We therefore measured MBC to reflect the dynamics of soil microbial biomass. MBC was estimated using the chloroform fumigation-extraction method^67^. The pH of a soil-water (1:2.5 w/v) suspension was measured using a pH meter (FE20, Mettler Toledo, Switzerland) after shaking for 30 min. Soil organic carbon (SOC) and TN were determined using an elemental analyzer (Elementar Vario EL III, Germany). AP in the soil was extracted with sodium bicarbonate and determined using the molybdenum blue method^68^. The soil KCl-extractable NO_3_^−^ and NH_4_^+^ were determined by extraction with 2 M KCl, followed by steam distillation and titration^69^.

### DNA extraction and library construction

Microbial DNA was extracted from the soil samples (0.25 g of wet weight) using an Ezup Column Soil DNA Purification Kit (Sangon Biotech, Shanghai, China) according to the manufacturer’s instructions. Total DNA was evaluated on a 1.0% agarose gel, and the DNA concentration and quality (A260/A280) of the extracts were estimated visually using a NanoDrop ND-1000 UV-V spectrophotometer (Thermo Scientific, Rockwood, TN, USA).

### 16S rRNA gene amplification and sequencing

The V4 hypervariable region of the 16S rRNA gene was amplified from each soil sample using the PCR primers 515F (5′-GTGCCAGCMGCCGCGGTAA-3′) and 806R (5′-GGACTACHVGGGTWTCTAAT-3′) and a sample tagging approach^70^,^71^. PCRswere performed in 30-μL reactions with 15 μL of Phusion® High-Fidelity PCR Master Mix (New England BioLabs, Ipswich, MA, USA), 0.2 μM forward and reverse primers, and approximately 10 ng of template DNA. Thermal cycling conditions consisted of initial denaturation at 98 °C for 1 min; 30 cycles of denaturation at 98 °C for 10 s, annealing at 50 °C for 30 s, and elongation at 72 °C for 30 s; and a final extension at 72 °C for 5 min. PCR products were purified with a Gene JET Gel Extraction Kit (Thermo Scientific) and combined in equimolar ratios with the quantitative DNA binding method to create a DNA pool that was subsequently used for sequencing from the adaptor. High-throughput sequencing of the 16S rRNA tag-encoded gene was performed on the Illumina MiSeq platform at Novogene (Beijing, China).

### Bioinformatics analysis

Sequencing reads were assigned to each sample according to the unique barcode tags. Sequences were analyzed with the QIIME (Quantitative Insights Into Microbial Ecology) software package and UPARSE pipeline^70^. The reads were first filtered by QIIME quality filters. The default settings for Illumina processing in QIIME were used. Then, the UPARSE pipeline was used to discern OTUs with 97% identity. For each OTU, a representative sequence was selected and a taxonomic group was assigned using the Ribosomal Database Project (RDP) classifier. The species richness of each sample was estimated by rarefaction analysis; the Chao1 index of each library was determined as described previously^72^.

### Statistical analysis

One-way analysis of variance (ANOVA) including post-hoc correction for multiple comparisons using the Bonferroni method was used to determine the statistical significance of differences in MBC and the Chao1 index for each sampling event among the four experimental treatments in the two plantation management schemes. Two-way ANOVA was performed to assess the combined effect of N deposition and management practices on microbial biomass and diversity. The Pearson correlation coefficient between soil properties and α-diversity was also calculated. Analyses were conducted using SPSS (Statistical Package for the Social Sciences) 18.0 for Windows (SPSS Inc., Chicago, Illinois, USA).

QIIME was used to calculate the weighted UniFrac. PCoA and UPGMA (unweighted pair group method with arithmetic mean) clustering were conducted on the weighted UniFrac based on a protocol published previously^73^. In addition, a CCA was performed to identify the abiotic factors with the most impact on bacterial community composition, and these results were used to construct a soil property matrix for variation partitioning analysis in R v.2.8.1 with the vegan package.

## Additional Information

**How to cite this article**: Li, Q. *et al*. Nitrogen deposition and management practices increase soil microbial biomass carbon but decrease diversity in Moso bamboo plantations. *Sci. Rep.* **6**, 28235; doi: 10.1038/srep28235 (2016).

## Figures and Tables

**Table 1:** Pearson’s correlation coefficients between soil properties and soil microbial community indicators.

	OTUs	Chao1	Shannon	MBC
pH	0.844**	0.785**	0.588**	−0.553**
SOC	−0.222	−0.219	−0.172	0.169
TN	−0.475*	−0.403	−0.299	−0.079
C/N	0.348	0.262	0.14	0.095
AP	−0.163	−0.192	−0.164	−0.018
NO_3_^−^	−0.644**	−0.564**	−0.404	−0.018
NH_4_^+^	−0.661**	−0.502*	−0.392	−0.1

OTUs: operational taxonomic units (97% identity); MBC: microbial biomass carbon; SOC: soil organic carbon; TN: total nitrogen; AP: available phosphorus.

*P < 0.05, **P < 0.01.

**Table 2:** Two-way ANOVA indicating the effects of N addition and management type (intensive or conventional) on soil microbial biomass carbon.

Source of variation	SS	df	MS	F	*P*-value
N addition	2742792.2	3	914264.1	1574.6	<0.0001
Management type	861123.7	1	861123.7	1483.0	<0.0001
Interaction	1264365.6	3	421455.2	725.8	<0.0001
Within	9290.4	16	580.7		
Total	4877572.0	23			

SS: sum-of-squares; df: degrees of freedom; MS: mean square.

**Table 3:** Two-way ANOVA indicating the effects of N addition and management type (intensive or conventional) on the Chao 1 index of soil microbial community composition.

Source of variation	SS	df	MS	F	*P*-value
N addition	1295767.9	3	431922.6	9.4	0.0008
Management type	8005801.7	1	8005802	175.2	<0.0001
Interaction	840578.7	3	280192.9	6.1	0.006
Within	730924.5	16	45682.8		
Total	10873072.9	23			

SS: sum-of-squares; df: degrees of freedom; MS: mean square.
